# Obesity-Driven Deficiencies of Specialized Pro-resolving Mediators May Drive Adverse Outcomes During SARS-CoV-2 Infection

**DOI:** 10.3389/fimmu.2020.01997

**Published:** 2020-08-11

**Authors:** Anandita Pal, Kymberly M. Gowdy, Kenneth J. Oestreich, Melinda Beck, Saame Raza Shaikh

**Affiliations:** ^1^Department of Nutrition, Gillings School of Global Public Health and School of Medicine, The University of North Carolina at Chapel Hill, Chapel Hill, NC, United States; ^2^Division of Pulmonary, Critical Care and Sleep Medicine, The Ohio State University Wexner Medical Center, Davis Heart and Lung Research Institute, Columbus, OH, United States; ^3^Department of Microbial Infection and Immunity, The Ohio State University College of Medicine and Wexner Medical Center, Columbus, OH, United States

**Keywords:** COVID-19, resolvins, protectins, maresins, lipoxins, antibodies

## Abstract

Obesity is a major independent risk factor for increased morbidity and mortality upon infection with Severe Acute Respiratory Syndrome Coronavirus (SARS-CoV-2), which is responsible for the current coronavirus disease pandemic (COVID-19). Therefore, there is a critical need to identify underlying metabolic factors associated with obesity that could be contributing toward increased susceptibility to SARS-CoV-2 in this vulnerable population. Here, we focus on the critical role of potent endogenous lipid metabolites known as specialized pro-resolving mediators (SPMs) that are synthesized from polyunsaturated fatty acids. SPMs are generated during the transition of inflammation to resolution and have a vital role in directing damaged tissues to homeostasis; furthermore, SPMs display anti-viral activity in the context of influenza infection without being immunosuppressive. We cover evidence from rodent and human studies to show that obesity, and its co-morbidities, induce a signature of SPM deficiency across immunometabolic tissues. We further discuss how the effects of obesity upon SARS-CoV-2 infection are likely exacerbated with environmental exposures that promote chronic pulmonary inflammation and augment SPM deficits. Finally, we highlight potential approaches to overcome the loss of SPMs using dietary and pharmacological interventions. Collectively, this mini-review underscores the need for mechanistic studies on how SPM deficiencies driven by obesity and environmental exposures may exacerbate the response to SARS-CoV-2.

## Introduction

Obesity is an independent risk factor for increased morbidity and mortality upon infection with the Severe Acute Respiratory Syndrome Coronavirus 2 (SARS-CoV-2) responsible for the current COVID-19 pandemic. Several studies underscore the notion that obesity, in addition to a range of other co-morbidities and dietary factors, may increase the risk for SARS-CoV-2 (1–10). As an example, in a study from Mexico, the odds of having COVID-19 among obese patients with a BMI > 30 kg/m^2^ was 61% higher than that of control non-obese patients (1). Generally, amongst patients with symptoms, those with severe or critical conditions had much higher BMI and prevalence of obesity than the normal population or COVID-19 negative patients (2–10). One study used the UK Biobank data (*n* = 285,817) to show that obesity almost doubled the risk of infection, adjusted for age, sex, ethnicity and socioeconomic status (9). Thus, it is clear that obesity results in a higher risk of increased severity of infection with SARS-CoV-2. These findings mirror influenza infection, as obesity also independently increases risk for influenza severity and death (11).

The high rate of obesity worldwide (e.g., in the U.S. over 40% of the adult population is obese) combined with the enhanced morbidity and mortality in obese individuals from infection with SARS-CoV-2 represents a public health emergency. Therefore, there is a critical need to identify the underlying factors by which obese patients are at high risk of infection and complications with SARS-CoV-2. In this mini-review, we focus on a unique aspect of fatty acid metabolism that may provide a link between obesity and immune dysregulation to SARS-CoV-2 infection. These significant insights could evoke new areas of investigation at a mechanistic level and ultimately therapeutic strategies for this vulnerable population.

## Metabolites of the Specialized Pro-Resolving Mediator Family Are Critical in the Resolution of Viral Infection Through Multiple Mechanisms

**Figure 1:**
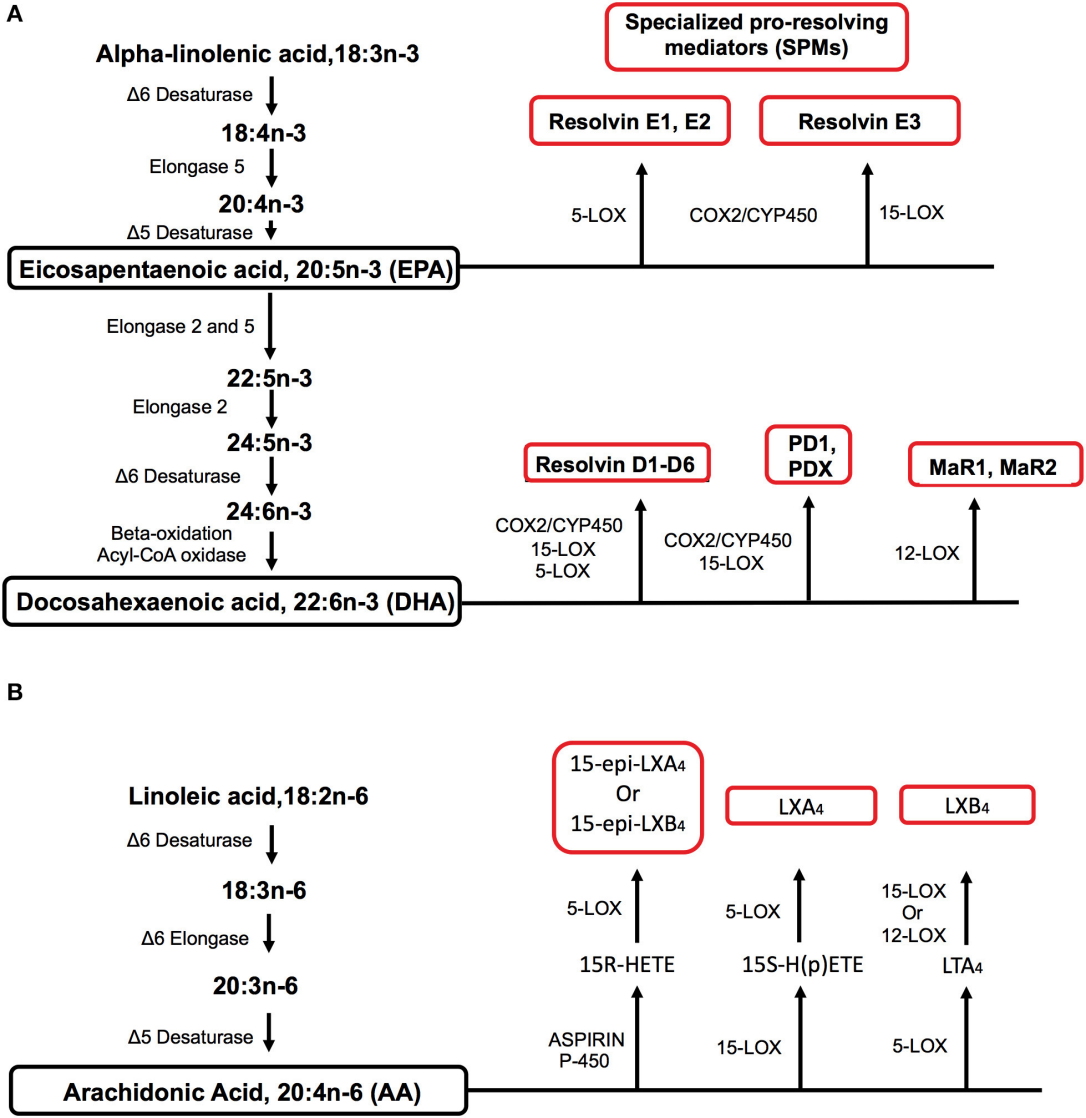
Metabolic pathways by which specialized pro-resolving mediators (SPMs) are synthesized from polyunsaturated fatty acids (PUFA). **(A)** EPA and DHA are long-chain n-3 PUFAs that serve as precursors for the biosynthesis of SPMs of the resolvin, protectin, and maresin families through the use of differing enzymes. EPA and DHA can be synthesized from the essential short-chain n-3 PUFA known as alpha-linolenic acid. **(B)** The biosynthesis of lipoxins from the n-6 PUFA arachidonic acid. Arachidonic acid can be synthesized from the essential n-6 PUFA linoleic acid. Key enzymes for fatty acid elongation and desaturation in addition to SPM biosynthesis are indicated for the n-3 and n-6 PUFA pathways. For simplicity, the biosynthesis of all SPM intermediates is not shown for the n-3 and n-6 pathways.

A wide range of metabolic factors contribute toward impaired innate and adaptive immunity in obesity. Here, we discuss the role of fatty acid-derived metabolites belonging to the specialized pro-resolving mediator (SPM) family. These potent lipid autacoids known as resolvins, protectins, maresins, and lipoxins are synthesized during the transition of inflammation to resolution and are critical for turning damaged tissue to homeostasis (12). SPMs are predominately synthesized from the n-3 polyunsaturated fatty acids (PUFA) known as eicosapentaenoic (EPA) and docosahexaenoic (DHA) acids (Figure 1A). Some SPMs are also synthesized from arachidonic acid, an n-6 PUFA (Figure 1B). For further details on these metabolites and their immunoresolvants properties, we refer the reader to elegant reviews from Serhan et al. (12, 13).

There is strong literature to support a role for SPMs in improving outcomes upon bacterial, parasitic, and viral infections (14, 15). To exemplify, the DHA-derived SPM known as protectin DX (PDX), an isomer of protectin D1 (PD1), enhanced mouse survival upon lethal H5N1 infection including under conditions where antiviral drugs failed to confer protection (16, 17). Mechanistically, PDX inhibited viral replication by targeting the nuclear export machinery for viral transcripts. PDX specifically blocked viral transcripts from being transported to NXF1, an mRNA transporter. Furthermore, pulmonary PDX levels were lowered upon influenza infection and were dependent on 12/15-lipoxygenase activity. These effects were unique to PDX as other PUFA-derived metabolites did not confer any improvement in survival.

Another study suggested that metabolites of the DHA-derived SPM family have utility as adjuvants for influenza vaccination. The SPM precursor 17-hydroxydocosahexaenoic acid (17-HDHA) increased antibody levels and improved survival upon pH1N1 influenza vaccination and infection in lean mice by promoting B cell differentiation toward the formation of CD138^+^ long-lived antibody secreting cells (18). At a molecular level, this was driven by 17-HDHA upregulating the expression of key transcription factors including Blimp-1, the master regulator of B cell differentiation toward antibody secreting plasma cells. Similarly, administration of dietary DHA ethyl esters, the parent compound of DHA-derived SPMs, also boost antibody levels of obese mice (19, 20). DHA improved antibody levels upon influenza infection by increasing the concentration of 14-hydroxydocosahexaenoic acid (14-HDHA), which in turn drove the formation of long-lived CD138^+^ antibody secreting cells (19). Therefore, these studies suggest that SPMs have a role in controlling influenza infection through differing mechanisms including improving aspects of humoral immunity. Furthermore, there is also *in vitro* evidence that the n-6 PUFA-derived SPM known as lipoxin B4 can stimulate antigen-specific IgG production from memory B cells in subjects that were vaccinated for influenza (21). In this case, lipoxin B4 upregulated the expression of Blimp-1 and XBP1 to increase the abundance of memory B cells.

The effects of SPMs are not just limited to influenza virus. For instance, aspirin-triggered resolvin D1 is reported to have anti-inflammatory effects on murine ocular inflammation driven by infection with herpes simplex virus (22). In addition, aspirin triggered resolvin D1 can clear mouse bacterial infections such as pulmonary pneumonia, which can lower the need for antibiotics (23, 24).

The cellular targets of SPMs in the context of viral infection and obesity are emerging. There is strong evidence for the role of SPMs in controlling chronic inflammation in obesity by targeting monocyte and macrophage polarization (25). This is particularly relevant for COVID-19 as adipose tissue presumably expresses high levels of the human angiotensin converting enzyme (ACE2), the receptor for SARS-CoV-2. ACE2 expression levels are likely higher in adipose tissue of the obese compared to the lungs, suggesting that adipose tissue may be a major target for SARS-CoV-2 (26). As described above, there is strong evidence on how SPMs drive B cell differentiation toward long-lived antibody secreting cells. However, it is unclear how SPMs influence other aspects of humoral immunity to promote antibody production. For instance, the abundance of T follicular helper cells, which are required to promote B cell activation and germinal center formation, is lowered in obesity (27). It remains unclear if SPMs could be targeting the abundance of these cells to improve germinal center formation and function. In addition, obesity impairs pulmonary outcomes upon influenza infection, including lung inflammation characterized by dysregulated memory CD8^+^ T cell metabolism (28). Given evidence to show that SPMs can control T cell differentiation and function, there is a need to understand the mechanisms by which SPMs may control the abundance and function of pulmonary T cell populations (29).

## Obesity Promotes a Signature of SPM Deficiency

There is evidence that obesity generally drives a unique signature of SPM deficiency (19, 30–37). Table 1 summarizes the results of these studies. To exemplify, obese mice compared to lean controls display a rapid reduction in DHA-derived SPM precursors and SPMs in white adipose tissue within 4 days of consuming a high fat diet (37). Others have also reported a reduction of not only DHA-derived SPMs but also metabolites from the EPA pathway upon long term consumption of obesogenic diets in white adipose tissue and liver, which are central in driving complications of obesity (30, 32, 34, 42). As described below, these deficiencies can be overcome through dietary administration of EPA- or DHA-enriched marine oils. On the contrary, one study demonstrated that in a model of liver steatosis, select SPMs were elevated, which may be due to an attempt to lower chronic inflammation (38). However, in this study, the liver content of EPA and DHA, the parent fatty acids of SPMs, were lower in obese mice relative to lean controls.

**Table 1:** Summary of the effects of obesity, diabetes, and weight loss on SPM levels across tissues of humans and mice.

**Model system**	**Tissue/cells**	**SPM precursors/SPMs**	**References**
Obese humans	Adipose tissue	The ratio of SPMs to leukotrienes and prostaglandins was significantly lowered in obese compared to lean individuals	(30)
Obese humans	Plasma & leukocytes	14-HDHA, 17-HDHA, 18-HEPE and 15-LXA_4_ levels were reduced in the plasma of obese compared to lean individuals. Leukocytes from obese individuals also had significantly lower levels of 17-HDHA and 18-HEPE	(31)
C57BL/6 mice	Adipose tissue	RvD1, PD1, 17-HDHA, 14-HDHA, and 18-HEPE levels were lower compared to lean mice	(32)
C57BL/6J mice	Spleen	PDX was lowered compared to lean controls	(19)
C57BL/6J mice	Spleen and bone marrow	14-HDHA, 17-HDHA and PDX were lower in obese male but not female mice. 14-HDHA was lowered in the bone marrow of obese male but not female mice	(33)
C57BL/6 mice	Adipose tissue and liver	15R-LXA_4_ increased in the adipose tissue of obese mice. 18-HEPE decreased in adipose and liver of obese mice	(34)
C57BL/6J mice	Adipose tissue macrophages	RvE1, RvE2, RvD2, RvD3, RvD5 levels were significantly reduced and RvD6 was significantly increased in obese mice	(35)
Swiss mice	Hypothalamus	Hypothalamic RvD2 is reduced in obese mice	(36)
C57BL6 and ob/ob mice	Adipose tissue	Adipose levels of 17-HDHA and PD1 are lowered in obese mice	(37)
C57BL/6J mice	Liver steatosis	Levels of liver RvE1, RvE2, RvD1 and RvD2 are increased compared to controls; EPA and DHA levels in the liver are lower in obese mice	(38)
*db/db* mice	Cutaneous wounds	17-HDHA, 14-HDHA and 4-HDHA levels were lower in the wounds of db/db mice	(39)
*db/db* mice	Adipose tissue	17-HDHA and PD1 were reduced and 18-HEPE was increased	(37)
Humans with and without out type 2 diabetes	Plasma	MaR1 levels are lowered in type 2 diabetic patients compared to controls. Diabetics with foot ulcers had a further reduction in maresin levels compared to controls and type 2 diabetics.	(40)
Humans with the metabolic syndrome and weight loss	Neutrophils	Metabolic syndrome patients who lost weight in a weight loss program had a 2-fold increase in RvE1 compared to those participants who were in the weight maintenance group and did not lose weight	(41)

SPM deficiencies are not just limited to adipose tissue and liver. When mice were fed a western diet, there was a significant loss of PDX in the spleen, which was reversed upon administration of DHA ethyl esters in the diet (19). A significant reduction of 14-HDHA, 17-HDHA, and PDX was also reported in mice consuming a high fat diet with a modest effect on 14-HDHA in the bone marrow (33). The effects were evident in male but not female obese C57BL/6J mice, suggesting sex differences in SPM deficiencies. In support of this notion, it is known that synthesis of DHA is higher in women than men (43). The notion of sex-differences in SPM metabolism is also consistent with a human study that showed females were protected from endothelial impairments driven by inflammation due to elevated levels of SPMs compared to males (44). The sex-differences are intriguing, as data on COVID-19 prevalence shows that males are disproportionally at higher risk for becoming infected than females across all ages (45).

Studies with human samples have validated murine studies by demonstrating that obese humans compared to lean controls display deficiencies of key SPM precursors in circulation. A major finding was that leukocytes isolated from obese patients had reduced levels of 17-HDHA and an unbalanced formation of DHA-derived resolvins along with an increased production of the potent chemokine leukotriene B_4_ (31). This study found impaired activity of 15-lipoxygenase, a key enzyme required for SPM biosynthesis to be the cause of the deficiency. Interestingly, the impairment was not due to reduced cellular uptake of DHA, consistent with rodent studies that show no impairment in DHA levels (33). Furthermore, when leukocytes were treated *in vitro* with 17-HDHA, the biosynthesis of downstream metabolites was rescued, demonstrating 15-lipoxygenase to be a potential therapeutic target for improving circulating levels of SPMs (31).

The observations on SPM deficiencies with obesity are generally consistent with models of type 2 diabetes, a major co-morbidity of obesity (Table 1). For instance, in wounds of *db/db* mice, select SPMs were lowered relative to littermate controls (39). In another study, 17-HDHA and PD1 were decreased in white adipose tissue of *db/db* mice, consistent with studies using diet-induced obese mice, although 18-HEPE levels were elevated compared to controls (37). In type 2 diabetic subjects, circulating maresin 1 (MaR1) levels were decreased compared to controls; furthermore, MaR1 was further decreased in those type 2 diabetics with foot ulcers (40). MaR1 is of significance given its role in regulating murine insulin sensitivity and adipose tissue inflammation in models of genetic and diet-induced obesity (46). Finally, a recent study showed weight loss elevated RvE1 levels in human subjects with metabolic syndrome (41), suggesting that the effects of obesity on SPMs could be potentially reversed through weight loss (Table 1).

## Obese Individuals Have Increased Susceptibility to Environmental Exposures That Drive a State of SPM Deficiency

Recent studies have noted that individuals living in areas with higher levels of ambient air pollution are at a higher mortality risk from COVID-19 (47, 48). This was also noted with previous SARS pandemics (49). Obese individuals are uniquely susceptible to environmental exposures and it is currently unknown whether there is a higher rate of mortality from COVID-19 in obese patients that live in areas with increased air pollution. Epidemiological studies have indicated an association between obesity and air pollution (50, 51). Studies of obese humans and animal models have demonstrated a greater decrement in pulmonary function after exposure to the criteria air pollutant ozone (O_3_), enhanced production of proinflammatory cytokines, and markers of oxidative stress (52, 53). It is currently unclear why obese individuals are more susceptible to the health effects of environmental exposures. However, experimental data have noted that obese mice and humans exposed to air pollutants have increased pulmonary and systemic TNFα, IL-17, markers of lung injury, and airspace neutrophilia (54).

In addition to increased inflammation, acute exposure to O_3_ significantly reduces pulmonary and systemic DHA-derived SPM precursors and SPMs (55). Treatment of mice with 17-HDHA, 14-HDHA, and PDX significantly decreased O_3_-induced pulmonary inflammation (55). This suppression of SPM production was also noted in a murine model of nanotoxicity wherein obese mice exposed to nanoparticles had a significant suppression in pulmonary expression of 5-lipoxygenase and 12/15-lipoxygenase and the production of EPA- and DHA- derived SPMs (56). Taken together, these data suggest that the susceptibility of obese individuals to environmental lung diseases may drive an altered pulmonary immune response and a state of SPM deficiency that increases the morbidity and mortality to respiratory infections, including COVID-19.

## Discussion

Given that SPM deficiencies in obesity are potentially contributing toward poor outcomes upon SARS-CoV-2 infection, administration of SPMs may be beneficial (57). This hypothesis assumes that SPMs would target key mechanisms by which SARS-CoV-2 drives an uncontrolled and dysregulated pulmonary response. SARS-CoV-2 can drive a cytokine storm, which may be a potential target for intervention as SPMs are known to have dual anti-inflammatory and pro-resolving properties including restricting excessive immune cell infiltration (12, 58). For instance, TNF-α, IL-6, IL-1β, IL-8, IL-12, monocyte chemoattractant protein 1 (MCP1), interferon-gamma inducible protein (IP10) and macrophage inflammatory protein 1A (MIP1A) have been implicated in driving complications associated with SARS-CoV-2 (59). Furthermore, uncontrolled infiltration of immune cells into the lungs, due to excessive reactive oxygen species and secretion of proteases promote pulmonary destruction and thereby lower blood oxygen upon SARS-CoV-2 infection (60). Thus, SPMs or their parent compounds may have utility in improving pulmonary cytokine production and recruitment of pulmonary immune cells upon infection. In support of this notion, in a mouse model of infection with non-typeable *haemophilus influenzae*, the aspirin triggered RvD1 decreased the concentration of pulmonary TNFα and IL-6 in addition to driving the clearance of macrophages (61).

There are several approaches that could increase levels of SPMs. One is through dietary intervention in which the parent compounds of SPMs, notably EPA and DHA, can be delivered as either over-the-counter supplements or as prescription supplements such as Lovaza, Vascepa, and Epanova. It is important to note that over-the-counter formulations of these fatty acids are not the same as prescriptions due to differences in dose, purity, and composition of the fatty acids. Nevertheless, a recent study showed that an SPM precursor containing marine oil strongly upregulated SPMs of the EPA and DHA series within hours of administration accompanied by enhanced neutrophil and monocyte phagocytosis of bacteria (62). However, a major limitation of this approach is that dietary EPA and DHA may not be as potent as direct intervention with SPMs (12). A more directed approach is to deliver SPMs rather than the parent compounds although the mode of delivery remains to be established. One recent study showed that SPMs were delivered using nanoparticles in a model of intestinal wound healing, which led to activation of pro-repair pathways in the colonic mucosa (63). Furthermore, changes in dietary patterns may be another viable option. The Western diet is associated with impaired pulmonary outcomes and a shift toward a Mediterranean diet may prevent a deficiency of SPMs (64).

An additional consideration is the potential role of n-6 PUFAs on outcomes related to SARS-CoV-2 infection. N-6 PUFAs are highly abundant in the western diet and there is some suggestion that select n-6 PUFAs such as linoleic acid could be driving SPM deficiencies due to competition between the n-6 and n-3 fatty acids for specific enzymes that control SPM biosynthesis (65, 66). This is particularly important to consider given that parenteral nutrition in a hospital setting is enriched in n-6 PUFA-enriched oils (67). Thus, increasing n-3 PUFA levels alone may not be enough to increase downstream SPMs in the obese but could require changes in the intake of n-6 PUFAs. Of course, n-6 PUFAs themselves are also critical for synthesis of SPMs such as lipoxins (12). Thus, additional studies on the complex relationship between dietary n-6 and n-3 PUFAs with downstream SPM biosynthesis, particularly in the context of viral infection are essential. Overall, there is no current evidence to support changes in dietary PUFA intake for improving outcomes upon SARS-CoV-2 infection, but is an important area of investigation at the pre-clinical and clinical level.

Finally, our understanding of the mechanisms by which SARS-CoV-2 exerts its effects are just emerging (60), although how the virus impairs outcomes in obese individuals currently remains unknown. There is no evidence for a role for SPMs in controlling the host's response upon SARS-CoV-2 infection. Therefore, there is a critical need to evaluate and understand the kinetics of SPM biosynthesis in human and animal models of obesity during SARS-CoV-2 infection using mass spectrometry-based lipidomics. Supporting experiments with gain and loss of function approaches in animal models are also required to establish that SPM deficiencies in obesity exacerbate the response to the infection. It is also important to consider the host genetic profile (34), which could be a major consideration in developing dietary or pharmacological approaches to overcoming SPM deficiencies and improving outcomes to SARS-CoV-2 for the obese.

## Conclusion

In summary, SPMs are key players in inflammation resolution and the infectious response. Deficiencies in SPMs, driven by obesity, its co-morbidities, and chronic pulmonary environmental exposures, could exacerbate the SARS-CoV-2 induced morbidities and mortalities. Thus, there is an urgency for mechanistic studies on SPMs in the context of obesity and its co-morbidities upon SARS-CoV-2 infection. Ultimately, targeting SPM deficiencies through dietary and pharmacological interventions may be a therapeutic approach worth investigating in order to decrease the morbidity and mortality in response to SARS-CoV-2 infection in a highly vulnerable and metabolically impaired population.

## Author Contributions

AP and KG wrote the manuscript. KO, MB, and SS wrote parts of the manuscript. SS assumes responsibility for the work. All authors contributed to the article and approved the submitted version.

## Conflict of Interest

The authors declare that the research was conducted in the absence of any commercial or financial relationships that could be construed as a potential conflict of interest.
